# The Automatic External Cardioverter-Defibrillator

**Published:** 2004-07-01

**Authors:** Antoni Martínez-Rubio, Gonzalo Barón-Esquivias

**Affiliations:** * Departments of Cardiology . Hosp. de Sabadell (Fund. Univ. Parc Tauli), Sabadell, Barcelona, Spain; † University Hospital Virgen del Rocio, Seville, Spain

**Keywords:** cardiac arrest, cost-effective, defibrillation, ventricular arrhythmias, ventricular fibrillation and ventricular tachycardia

## Abstract

In-hospital cardiac arrest remains a major problem but new technologies allowing fully automatic external defibrillation are available. These technologies allow the concept of “external therapeutic monitoring” of lethal arrhythmias. Since early defibrillation improves outcome by decreasing morbidity and mortality, the use of this device should improve the outcome of in-hospital cardiac arrest victims. Furthermore, the use of these devices could allow safe monitoring and treatment of patients at risk of cardiac arrest who not necessarily must be in conventional monitoring units (Intensive or Coronary Care Units) saving costs with a more meaningful use of resources. The capability to provide early defibrillation within any patient-care areas should be considered as an obligation (“standard of care”) of the modern hospital.

## Introduction

Cardiovascular disease is the major cause of death in the majority of countries [1,2]. In developed countries (e.g. United States), nearly the half of these deaths are unexpected and sudden [3,4]. Although the broad majority of sudden cardiac deaths occur outside the hospital and very poor survival rates have been reported, in-hospital cardiac arrest remains a major problem. The aim of this article is to review the actual knowledge of the utility of “fully automatic” external defibrillators for treatment of in-hospital cardiac arrest. The name “automated” external defibrillator (AED) must be distinguished from fully “automatic” external defibrillator. The automatic device is able to deliver a shock without intervention from an operator whereas the automated device only “advises” by a voice or a text to deliver the energy. Thus, the automated device requires operator intervention.

## Physiological considerations of cardiac arrest

Cardiopulmonary resuscitation maneuvers can sustain a patient for a certain time but is unlikely to restore an organized rhythm of the heart. To achieve this goal defibrillation and advanced cardiovascular care are necessary, which must be administered in the shortest possible interval after the arrhythmic event. Furthermore, the risk of ischemia and the defibrillation threshold increase with arrhythmia duration [5]. The time to defibrillation is the single most important determinant of survival from cardiac arrest [6-17]. In addition, ventricular fibrillation (VF) tends to evolve to asystole within few minutes [18-27]. Thus, survival rates after VF decrease approximately 7% to 10% with every minute that defibrillation is delayed [18-24]. In addition, the cerebral cortex is irreversibly damaged if cardiac arrest is not quickly interrupted [18,28-30]. Because of these facts, individual physicians as well as the American Heart Association, the American College of Emergency Physicians, the European Resuscitation Council and others have advocated the widespread dissemination of defibrillators for decreasing mortality and morbidity for victims of cardiac arrest [31,34].

## In-hospital cardiac arrest

### The classical reality

Sustained ventricular tachycardia (VT) and VF, even in hospitalized patients, are major causes of morbidity and mortality [28,35-37]. In the best setting (monitoring wards), continuous ECG monitoring allows identification of lethal arrhythmias and alarm systems alert nursing and medical staff. However, a time delay between the arrhythmic event and human intervention obviously exists. This time delay may be prolonged in certain circumstances even in monitoring wards. In addition, response time intervals for in-hospital resuscitation events are often inaccurate and must be corrected before documented times to defibrillation can be considered reliable [34].

Although major evidence exists supporting the need for rapid defibrillation and important advances in out-of-hospital cardiac arrest treatment have been achieved (e.g. out-of hospital cardiac arrest quick response programs) [15,16,34,38-43], in-hospital cardiac arrest is still a major problem without significant advances (e.g. changing strategies) during the last 30 years. This leads to important mortality, morbidity and social as well as economic costs [28,35-37]. The scarcity of data [28,44] related to deployment of AEDs in hospitals and its impact on patient outcome reflects the very limited existence of in-hospital early defibrillation programs.

However, as stated in major guidelines, early defibrillation is a high-priority goal in out-of hospital as well as in-hospital cardiac arrest [31-34]. Clearly the earlier defibrillation occurs, the better the prognosis in adults and children [18-21,23-25,27,45-47].

The capability to provide early defibrillation within any patient-care areas should be considered as an obligation (“standard of care”) of the modern hospital. Furthermore, cardiac arrests often occur outside monitored areas. Recently, Herlitz et al [28 ]. reported that out of 557 patients suffering in-hospital cardiac arrest, only 292 patients (53%) were in monitored wards and from those only 43.2% of the patients could be discharged alive. They reported that the median interval between collapse and first defibrillation was 1 minute in monitored wards and 5 minutes in non-monitored wards. Only 31% of patients from non-monitored wards could be discharged alive and with a cerebral performance inferior to that of survivors of monitored wards. Other authors [48 ] present similar data showing better in-hospital survival for witnessed arrest (25%) than for non-witnessed arrest (7%) but, in addition, they report a disproportionately high incidence of non-witnessed arrests during the night (12AM to 6 AM) resulting in a very poor survival rate (0%). Cardiac arrest occurs in 4.8% of hospitalized patients because of acute myocardial infarction [49 ]. The survival rate to hospital discharge for these individuals was 29.4% [49 ]. Although ventricular tachycardia or fibrillation was documented in 34.7% of patients, only 47.5% of those survived to discharge [49 ]. Thus, the use of “automatic” defibrillators had probably improved outcome of those patients presenting in-hospital cardiac arrest. Furthermore, although survival to hospital discharge offers an objective evaluation point and is used in the broad majority of reports, several patients who survive a cardiac arrest present neurological damage, which is highly dependent of the response time to cardiac arrest [18 ,28,29 ]. Therefore, the neurological status should also be considered when reporting results of resuscitation procedures [30 ].

### The new reality

Very early response to lethal ventricular tachyarrhythmias is achievable now with fully “automatic” external defibrillators. As occurs with implantable cardioverter defibrillators, these devices may be individually programmed to intervene. Thus, as occurs with other medical management strategies (e.g. drugs prescription), physicians may and should decide under which circumstances and how the device will react in presence of arrhythmias.

In a multicenter European trial, Martinez-Rubio et al. [50 ] tested the safety and efficacy of an automatic external cardioverter-defibrillator [Powerheart®; Cardiac Science Inc., Irvine (California, USA)]. Subjects (n=117) included patients undergoing electrophysiologic testing (n=66; all with suspected/documented VT/VF or undergoing implantation of implantable cardioverter defibrillator) as well as patients in monitoring wards (n=51) at risk of cardiac arrest.  Patients with active implanted cardioverter-defibrillators were excluded from the study.

The automatic external cardioverter-defibrillator was connected to patients using self-adhesive electrodes.  Placement of the electrodes (either antero-posterior or sternal-apex) was at the discretion of the investigators.

The automatic external defibrillator was used for monitoring and treatment of patients in the coronary care units or intensive care units.  It was also used as a “rescue” defibrillator during ICD implantations. This device operates either in “advisory” or in “automatic” mode.  In advisory mode, it will detect the arrhythmia, alarm, charge the capacitors, and prompt the user to deliver the shock. The energy (maximum of 360J monophasic) and time delay (10s to 600s) for each shock could be programmed (up to 9 shocks per episode).  Shock delivery was non-committed such that therapy is automatically aborted should the rhythm spontaneously convert to a non-shockable rhythm.  In addition, the device algorithm featured a modulation domain function which allows to discriminate between ventricular and supraventricular rhythms.

**Figure 1:**
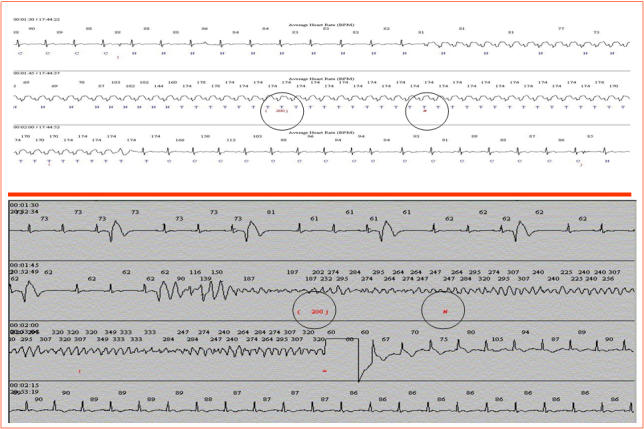
**Upper panel:** Recording of the automatic external cardioverter-defibrillator response to spontaneous monomorphic ventricular tachycardia. The device is programmed to detect the arrhythmia, to charge and to delay 1 minute the shock delivery. After 24 seconds and spontaneously, the tachycardia terminates. Observe that the device aborts the shock delivery.  The first circle indicates de beginning of charge (200 J) of the device. The second circle indicates the full charge of the device (“ready to shock”). Each line corresponds to 15 seconds of continuous ECG registration. **Lower panel:**Recording of the automatic external cardioverter-defibrillator response to spontaneous ventricular fibrillation in a patient with admitted because of recurrent syncope. The first circle indicates de beginning of charge (200 J) of the device. The second circle indicates the full charge of the device. Note that the device terminates the arrhythmia when applies the shock. Each line corresponds to 15 seconds of continuous ECG registration.

The performance of the device in detecting shockable and non-shockable rhythms was confirmed by review of the simultaneously recorded Holter data and the programmed parameters. A total of 1,240 hours of monitoring and 1,988 episodes of rhythm changes were documented.  The device detected ventricular arrhythmias with a sensitivity of 100% and specificity of 97.6%.  The mean response time to shock was 14.4 seconds [evaluated in those episodes (sustained) that were associated with a full capacitor charge cycle].  All false positives were caused by T-wave oversensing during ventricular pacing.  There were no complications or adverse events. There were a total of 35 sustained arrhythmic episodes treated by the device (12 in the EP lab and 23 in monitoring wards).  All 35 episodes were successfully (100% efficacy) converted to normal sinus rhythm with a first shock success of 94.3% (Figure 1).  This European study [50 ] confirms the initial experience presented by Mattioni et al [51 ] in a multicenter American trail. These authors reported a sensitivity and specificity of 100% and 99.4%, respectively, and a response time of 22 seconds [51 ].

Although some differences exist (e.g. sample size, hours of monitoring, software version, etc.) both studies [50,51 ] lead to the same conclusions. Thus, both groups (European and American) of investigators agree that the device is safe and highly effective in monitoring, detecting and treating spontaneous rhythms. Therefore, with its wide use a significant improvement in the treatment of in-hospital cardiac arrest should be expected. In addition, the now available version of the described automated external cardioverter-defibrillator has important improvements (compatibility to commercially available patient monitoring systems, biphasic wave forms, etc.).

## References

[R1] Murray CJL, López AD (1997). Alternative projections of mortality and disability by cause 1990-2020: Global Burden of Disease Study. Lancet.

[R2] Achutti A, Balaguer-Vintró I, Bayés de Luna A, Chockalingam A, Balaguer-Vintro I (1999). Impending global pandemic of cardiovascular disease.

[R3] Bayés de Luna A, Coumel P, Leclercq JF (1989). Ambulatory sudden death: mechanisms of production of fatal arrhythmia on the basis of data from 157 cases. Am Heart J.

[R4] Zipes DP, Wellens HJ (1998). Sudden Cardiac Death. Circulation.

[R5] Winkle RA, Mead RH, Ruder MA (1990). Effect of duration of ventricular fibrillation on defibrillation efficacy in humans. Circulation.

[R6] Cummins RO, Eisenberg MS, Bergner L (1984). Automatic external defibrillation: evaluations of its role in the home and in emergency medical services. Ann Emerg Med.

[R7] Cummins M, Eisenberg MS, Moore JE (1985). Automatic external defibrillators: clinical, training, psychological, and public health issues. Ann Emerg Med.

[R8] Atkins JM (1986). Emergency medical service systems in acute cardiac care: state of the art. Circulation.

[R9] Cummins RO, Eisenberg MS, Stults KR (1986). Automatic external defibrillators: clinical issues for cardiology. Circulation.

[R10] Eisenberg MS, Cummins RO (1986). Defibrillation performed by the emergency medical technician. Circulation.

[R11] Jacobs L (1986). Medical, legal, and social implications of automatic external defibrillators. Ann Emerg Med.

[R12] Cummins RO (1987). EMT-defibrillation: national guidelines for implementation. Am J Emerg Med.

[R13]  Newman MM (1987). The survival advantage: early defibrillation programs in the fire service. J Emerg Med Serv.

[R14] Ruskin JN (1988). Automatic external defibrillators and sudden cardiac death. N Engl J Med.

[R15] Sedgwick ML, Watson J, Dalziel K (1992). Efficacy of out of hospital defibrillation by ambulance technicians using automated external defibrillators: the Heartstart Scotland Project. Resuscitation.

[R16] Mols P, Beaucarne E, Bruyninx J (1994). Early defibrillation by EMTs: the Brussels experience. Resuscitation.

[R17] Stapczynski JS, Svenson JE, Stone CK (1997). Population density, automated external defibrillator use, and survival in rural cardiac arrest. Acad Emerg Med.

[R18] Weaver WD, Copass MK, Bufi D (1984). Improved neurologic recovery and survival after early defibrillation. Circulation.

[R19] Bachman JW, McDonald GS, O’Brien PC (1986). A study of out-of-hospital cardiac arrests in northeastern Minnesota. JAMA.

[R20] Cummins RO (1989). From concept to standard-of-care? Review of the clinical experience with automated external defibrillators. Ann Emerg Med.

[R21] Olson DW, LaRochelle J, Fark D (1989). EMT-defibrillation: the Wisconsin experience. Ann Emerg Med.

[R22] Eisenberg MS, Horwood BT, Cummins RO (1990). Cardiac arrest and resuscitation: a tale of 29 cities. Ann Emerg Med.

[R23] Eisenberg MS, Cummins RO, Damon S (1990). Survival rates from out-of-hospital cardiac arrest: recommendations for uniform definitions and data to report. Ann Emerg Med.

[R24] Larsen MP, Eisenberg MS, Cummins RO (1993). Predicting survival from out-of-hospital cardiac arrest: a graphic model. Ann Emerg Med.

[R25] Valenzuela TD, Spaite DW, Meislin HW (1993). Emergency vehicle intervals versus collapse-to-CPR and collapse-to-defibrillation intervals: monitoring emergency medical services system performance in sudden cardiac arrest. Ann Emerg Med.

[R26] Valenzuela TD, Roe DJ, Cretin S (1997). Estimating effectiveness of cardiac arrest interventions: a logistic regression survival model. Circulation.

[R27] Holmberg M, Holmberg S, Herlitz J (1998). Survival after cardiac arrest outside hospital in Sweden: Swedish Cardiac Arrest Registry. Resuscitation.

[R28] Herlitz J, Bang A, Aune S (2001). Characteristics and outcome among patients suffering in-hospital cardiac arrest in monitored and non-monitored areas. Resuscitation.

[R29] Jorgensen EO, Holm S (1999). The course of circulatory and cerebral recovery after circulatory arrest: influence of prearrest, arrest and post-arrest factors. Resuscitation.

[R30] Rosomoff HL, Kochanek PM, Clark R (1996). Resuscitation from severe brain trauma. Crit Care Med.

[R31] McDowell R, Krohmer J, Spaite DW (1993). American College of Emergency Physicians. Guidelines for implementation of early defibrillation/automated external defibrillator programs. Ann Emerg Med.

[R32] Kloeck W, Cummins RO, Chamberlain D (1997). Early defibrillation: an advisory statement from the Advanced Life Support Working Group of the International Liaison Committee on Resuscitation. Circulation.

[R33] Bossaert L, Handley A, Marsden A (1998). European Resuscitation Council guidelines for the use of automated external defibrillators by EMS providers and first responders: a statement from the Early Defibrillation Task Force, with contributions from the Working Groups on Basic and Advanced Life Support, and approved by the Executive Committee. Resuscitation.

[R34] (2000). Guidelines 2000 for Cardiopulmonary Resuscitation and Emergency Cardiovascular Care. The American Heart Association in Collaboration with the International Liaison Committee on Resuscitation. Part 4: The automated External Defibrillator. Key link in the Chain of Survival. Circulation.

[R35] Bedell SE, Delbanco TL, Cook EF (1983). Survival after cardiopulmonary resucitation in the hospital. N En J Med.

[R36] Kaye W, Mancini ME (1996). Improving outcome from cardiac arrest in the hospital with a reorganized and strengthened chain of survival.: an American view. Resuscitation.

[R37] Thel MC, O'Connor CM (1999). Cardiopulmonary resuscitation: historical perspective to recent investigations. Am Heart J.

[R38] O’Rourke ME, Donaldson E, Geddes JS (1997). An airline cardiac arrest program. Circulation.

[R39] Page RL, Hamdan MH, McKenas DK (1998). Defibrillation aboard a commercial aircraft. Circulation.

[R40] White RD, Asplin BR, Bugliosi TF (1996). High discharge survival rate after out-of-hospital ventricular fibrillation with rapid defibrillation by police and paramedics. Ann Emerg Med.

[R41] Karch SB, Graff J, Young S (1998). Response times and outcomes for cardiac arrests in Las Vegas casinos. Am J Emerg Med.

[R42] Mosesso VN, Davis EA, Auble TE (1998). Use of automated external defibrillators by police officers for treatment of out-of-hospital cardiac arrest. Ann Emerg Med.

[R43] Davis EA, McCrorry J, Mosesso VN (1999). Institution of a police automated external defibrillation program: concepts and practice. Prehosp Emerg Care.

[R44] Destro A, Marzaloni M, Sermasi S (1996). Automatic external defibrillators in the hospital as well?. Resuscitation.

[R45] Hickey RW, Cohen DM, Strausbaugh S (1995). Pediatric patients requiring CPR in the prehospital setting. Ann Emerg Med.

[R46] Mogayzel C, Quan L, Graves JR (1995). Out-of-hospital ventricular fibrillation in children and adolescents: causes and outcomes. Ann Emerg Med.

[R47] Losek JD, Hennes H, Glaeser P (1987). Prehospital care of the pulseless, nonbreathing pediatric patient. Am J Emerg Med.

[R48] Dumot JA, Burval DJ, Sprung J (2001). Outcome of adult cardiopulmonary resuscitations at a tertiary referral center including results of “limited” resuscitations. Archives of Internal Medicine.

[R49] Ornato JP, Peberdy MA, Tadler SC (2001). Factors associated with the occurrence of cardiac arrest during hospitalization for acute myocardial infarction in the Second National Registry of Myocardial Infarction in the US. Resuscitation.

[R50] Martinez-Rubio A, Kanaan N, Borggrefe M (2003). Advances for treating in-hospital cardiac arrest: safety and effectiveness of a new automatic external cardioverter-defibrillator. J Am Coll Cardiol.

[R51] Mattioni TA, Nademanee K, Brodsky M (1999). Initial clinical experience with a fully automatic in-hospital external cardioverter defibrillator. PACE.

